# Supplementary material for: Seroprevalence and Shifting Endemicities of Hepatitis A Virus Infection in Two Contrasting Geographical Areas in Indonesia
Source: Medicina (Kaunas). 2025 Apr 26;61(5):806. doi: 10.3390/medicina61050806 (PMC12112880; doi:10.3390/medicina61050806)
Supplement: Supplementary file 1 [file medicina-61-00806-s001.zip › Supplementary Table S1. Age-stratified sample size estimations, per geographical area.pdf]

Supplementary Table S1. Age-stratified sample size estimations, per geographical area

Age Group	Expected HAV Seroprevalence Prevalence	Sample size Needed n=	Safe Margin of error n=
1-2y	0.1	50	55
3-4y	0.1	50	55
5<10y	0.2	44	50
10-14y	0.2	44	50
15-19y	0.4	65	70
20-24	0.5	68	75
25-29	0.5	68	75
30-34	0.7	57	65
35-39	0.7	57	65
40-49	0.8	44	50
50+	0.9	25	30
**Total per 2 geographical areas**		**572**	**640**
**Total (country)**		**1144**	**1280**

Sample size calculation considered the age specific seroprevalence observed in Thailand[16] in 1990 and 2004. Sample size is calculated using the formula: $n = (Z^2 \text{ x } P(1 / P))/e^2$

Where:

- Z = value from standard normal distribution corresponding to desired confidence level (Z=1.64 for 90% CI)
- P is expected true proportion
- e is desired precision

With an absolute precision of 5% and 10% for prevalence estimates of 10% and more than 10% respectively, and confidence interval of 90% (well suited parameters for a descripted study using a high accurate diagnostic tool for HAV antibodies detection) and taking into account the population pyramid structure.
