# Supplementary material for: Seroprevalence and Shifting Endemicities of Hepatitis A Virus Infection in Two Contrasting Geographical Areas in Indonesia
Source: Medicina (Kaunas). 2025 Apr 26;61(5):806. doi: 10.3390/medicina61050806 (PMC12112880; doi:10.3390/medicina61050806)
Supplement: Supplementary file 1 [file medicina-61-00806-s001.zip › Supplementary Table S2. Interviewer-administered questionnaire (IAQ).pdf]

Supplementary Table S2. Interviewer-administered questionnaire (IAQ)*

**1**	Participant Study ID: ________	**7.** Date of Survey DD/MM/YYYY
**2**	Initials of participant : _ _ _	**8.** Name of the Survey taker:
**3**	Village/ city of residence:	
**4**	Area or residence: Urban/ Rural	
**5**	Date of Birth* DD/MM/YYYY	
**5a**	Does Date match with ID card 1. Yes 2. No	
**6**	Gender: 1. Male 2. Female	

<u>*To be read by the survey taker before starting the survey*</u>

Dear participant

Thanks for accepting to participate in this Epidemiological study. This questionnaire will register information related to Hepatitis A disease. This information in addition to the blood sample that you have donate to the study will help to understand better the behaviour of Hepatitis A in your community and will provide relevant information to Healthcare provider to take action for a better control of this disease. You are free to answer o not any of the questions of this questionnaire. Ones again thank you very much for your participation

Inclusion Criteria: All participants should fulfil all the following criteria, otherwise are not eligible to participate. *Please circle or tick, whenever applicable*

I1	Age 1-80 years at the time of enrolment.	Yes / No
I2	Being resident (living during the last 6months) in the selected geographic area	Yes / No
I3	Willing to participate and provide Informed consent (and attestation for minors according to local good research practices) by participant or parent and/ or legal guardian for children.	Yes / No

Exclusion Criteria: Any subject answering YES to any of the following criteria will be excluded from the study and could not continue the survey

I4	Known or suspected congenital or acquired immunodeficiency.	Yes / No
I5	Individuals diagnosed with Terminal or psychiatric illness	Yes / No
I6	Receipt of immune globulins, blood or blood-derived products in last 3 months.	Yes / No
I7	Attending to a health care institution due to gastrointestinal symptoms or icterical in the last 8 weeks	Yes / No
I8	Bleeding disorder, or receipt of anticoagulants in the 3 weeks preceding inclusion, contraindicating blood drawing.	Yes / No
I9	Child, parent or family member (living in the same household) already enrolled as participant in this study.	Yes / No
I10	Children in care – institutions or orphanages	Yes / No

**9**	Child / Individual eligible for participation in the study: 1. Yes 2. No

If yes, complete the rest of the form, if not acknowledge the time and interest to participate into the study and provide information leaflet of Hepatitis A disease.

Section A. Sociodemographic

Please use a circle to mark the answer given by the participant/parent

**A1**	Education of 12+ participant:<br>1. Primary School (6years)<br>2. Middle School (9 years)<br>3. High School (13 years)<br>4. Graduate/post-graduate (16+ years)<br>5. Illiterate<br>6. <6y (for participants <12y age)
**A2**	Occupation of participant:<br>1. Professional 2. Semi-professional 3. Clerical/shop owner<br>4. Skilled worker 5. Semi-skilled worker 6. Unskilled worker<br>7. Household duties 8. Unemployed (adults) 9.Attending School<br>10. Attending Garderie/Pre-School 11. At home (children)
**A3**	For all participants, What was the highest education attained from you parents of parents choose the option number as mentioned in A1<br>(a) Education of Father: 1- 2- 3- 4- 5- 6-<br>(b) Education of Mother: 1- 2- 3- 4- 5- 6-
**A4**	Total number of family members living in the same household: _ ________
**A5**	Total family monthly income __________________<br>*(local currency, please consider to include multiple selection accordingly to local scenario:*
**A6**	Regarding your households walls roof and floor are made of (please choose one)<br>○ Permanent building materials are used for walls, roof and floor<br>○ Natural/Traditional/ Rudimentary materials
**A7**	Do you have at home (you can tick more than one option<br>○ Fridge/Refrigerator<br>○ Bank account<br>○ Iron<br>○ Desktop/laptop<br>○ Radio<br>○ Sofa<br>○ Sewing machine
**A8**	How many habitable rooms your household has? _______________

Section B . Knowledge of disease

(Participants 14+ ***should answer by themselves*** *these Section of question in not otherwise specified)*

**B1**	Have you heard about a disease called Hepatitis A before?<br>*(If the answer is "No" then go to the Section C)*	1. Yes 2. No
**B2**	What kind of a disease is it?	1.Communicable<br>2.Non-Communicable<br>3. Do not know
**B3**	What is the main way of transmission?	1. By blood<br>2. By air<br>3. Sexually Transmitted

		4. By contaminated food/water<br>5. By mosquito bite
**B4**	Is there a vaccine to prevent Hepatitis A available in Indonesia?	1. Yes<br>2. No<br>3. Do not know
**B5**	What are the possible risk factor/ factors? (Multiple answers are allowed)	○ Use unclean toilets<br>○ Consume contaminated water/ food<br>○ Talking to ill<br>○ Sharing a room with an infected individual
**B6**	What are the possible symptoms of Hepatitis A?<br><br>a. Yellowish discoloration of eyes — Yes — No — Don't know<br>b. Abdominal pain — Yes — No — Don't know<br>c. Nasal bleeding — Yes — No — Don't know<br>d. Dark tea colour urine — Yes — No — Don't know<br>e. Numbness over extremities — Yes — No — Don't know<br>f. Fever — Yes — No — Don't know<br>g. Pale stools — Yes — No — Don't know	

Section C. Past Medical History of Hepatitis

(participants 14+ can answer this section questions with the assistance of their guardian)

**C1**<br><br>**C1a**<br><br>**C1b**<br>**C1c**<br>**C1d**	Have you (your child) ever been diagnosed with Hepatitis Disease?<br>1. Yes 2. No 3. Don't Know (DK)<br>(a) If Yes, what type? ____________, if No go to Question C2<br>(*Write the answer provided, if the answer is Don't Know, write down DK)*<br>(b) if Yes, were you living in the same household as today? 1. Yes 2. No<br>(c) if No to the last question, where were you living? (Village Name) ________________<br>(d) What was the Area of Residence of your household when got infected? 1. Urban 2. Rural
**C2a**<br><br>**C2b**	(a) Did you (your child) live the most during your first 5 years of life in the current village? 1. Yes 2. No<br>(b) What was the Area of Residence of your household at that time: 1. Urban 2. Rural
 C3a<br>**C3b**	Has anyone in your family got jaundice with fever in the past? 1. Yes 2. No<br>(a) If yes, who? ______________ (mother /father/siblings /others (specify))<br>(b) When (at least the year of occurrence. If more than one member affected record the earliest reported date)? MM/YEAR
**C4a**<br>**C4b**	Have you ever been vaccinated against Hepatitis A? 1. Yes 2. No<br>*Confirm the answer by checking vaccination card??* 1. Yes 2. No

Section D . Drinking Water Safety
(*participants 14+ can answer this section questions with the assistance of their guardian)*

**D1**	What is the main source of drinking-water for members of your household?<br>*(If answer is #10 go to D2, otherwise jump to D3)*<br><br>1. Piped water into dwelling<br>2. Piped water to yard/plot<br>3. Public tap/ standpipe<br>4. Tubewell/ bore-hole<br>5. Protected dug well<br>6. Unprotected dug well<br>7. Protected spring<br>8. Unprotected spring<br>9. Rainwater collection<br>10. Bottled water<br>11. Cart with small tank/drum<br>12. Tanker-truck<br>13. Surface water (river, dam, lake, pond, stream, canal, irrigation channels)<br>14. Other (specify) ______________
**D2**	You indicated that bottled water was your main source of drinking water, now I will like to ask you what is the main source of water used by your household **for other purposes, such as cooking and hand washing?**<br>1. Piped water into dwelling<br>2. Piped water to yard/plot<br>3. Public tap/ standpipe<br>4. Tubewell/ bore-hole<br>5. Protected dug well<br>6. Unprotected dug well<br>7. Protected spring<br>8. Unprotected spring<br>9. Rainwater collection<br>10. Bottled water<br>11. Cart with small tank/drum<br>12. Tanker-truck<br>13. Surface water (river, dam, lake, pond, stream, canal, irrigation channels)<br>14. Other (specify) ______________
**D3**	How long does it take to go there, get water, and come back?<br>#. of minutes ________________ ,<br>*(Ask for his/her best guess, otherwise please write down DK if participant don't know the answer to this question or 0 (zero) if water source is within the premises of the household)*
**D4**	Do you treat your water in any way to make it safer to drink? 1. Yes 2. No
**D5**	What do you usually do to the water to make it safer to drink?<br>Record all items mentioned<br>o Boil<br>o Add bleach/chlorine<br>o Strain it through a cloth<br>o Use a water filter (ceramic, sand, composite, etc.)<br>o Solar disinfection<br>o Let it stand and settle<br>o Other (specify)<br>o Don't Know
**D6**	What kind of toilet facility do members of your household usually use?<br>If "flush" or "pour flush" probe: Where does it Flush/pour flush to:<br><br>1. Piped sewer system<br>2. septic tank<br>3. pit latrine<br>4. elsewhere<br>5. unknown place/not sure/<br>6. Ventilated improved pit latrine (VIP)<br>7. Pit latrine with slab<br>8. Pit latrine without slab/open pit<br>9. Composting toilet<br>10. Hanging toilet/hanging latrine<br>11. No facilities or bush or field<br>12. Other (specify)

**D7**	How many households use this toilet facility? __________<br>*(please write down 0 (cero) if the toilet is not shared with any other household or DK if the participant don’t know an answer for this question*
**D8**	Can any member of the public use this toilet? 1. Yes 2. No 3. DK
**D9**	*This question applies to participants <4y age.*<br>*The* last time [name of youngest child] passed stools, what was done to dispose of the stools*?*<br>1. Child used toilet/latrine<br>2. Put/rinsed into toilet or latrine<br>3. Put/rinsed into drain or ditch<br>4. Thrown into garbage<br>5. Buried<br>6. Left in the open<br>7. Other (specify)__________<br>8. DK

Section E. Hygienic Food Intake

participants 14+ can answer this section questions with the assistance of their guardian

**E1**	Where do you prepare your food at home?	1. On the ground<br>2. multi-purpose table<br>3. Table exclusively set for cooking

Please answer the following questions based on what you have done during the past 7 days

		**Category**			
	**Question:**	**Never**	**Sometimes**	**Most of the time**	**Always**
**E2**	Did you get your main meals from home?	○	○	○	○
**E3**	Did you get your main meals from street?	○	○	○	○
**E4**	Did you wash your hands before handling food?	○	○	○	○
**E5**	Did you wash your hands before eating food?	○	○	○	○
**E6**	Did you wash your hands after defecation? *(practice of mother should be asked in case of younger children)*	○	○	○	○
**E7**	Was the kitchen which prepared the food you ate, free of insects and rodents?	○	○	○	○

*Designed by the participant Investigators for the purpose of this study and based on a previously validated one for Hepatitis A risk factors[16,19], the WHO/UNICEF core questions on drinking water, sanitation and hygiene for household surveys[20] and a recent systematic review on the risk factors of sporadic HAV infection[2].
