# Supplementary material for: Seroprevalence and Shifting Endemicities of Hepatitis A Virus Infection in Two Contrasting Geographical Areas in Indonesia
Source: Medicina (Kaunas). 2025 Apr 26;61(5):806. doi: 10.3390/medicina61050806 (PMC12112880; doi:10.3390/medicina61050806)
Supplement: Supplementary file 1 [file medicina-61-00806-s001.zip › Supplementary Table S3. Calculation of the Water_Sanitation, Assets, Maternal education, and Income (WAMI) index .pdf]

Supplementary Table S3. Calculation of the Water/Sanitation, Assets, Maternal education, and Income (WAMI) index

Index	Description	Range
Water/ sanitation	Using the World Health Organization's definitions of access to improved water and improved sanitation, households with access to improved water or improved sanitation are assigned a score of 4 for each. Households without access to improved water or improved sanitation are assigned a score of 0 for each. These scores were summed.	0-8
Assets	Eight priority assets were selected using random forests with HAZ as the outcome. For each asset, households were assigned a 1 if they had the asset and 0 if they did not have the asset. These scores were summed.	0-8
Maternal education	Each child's mother provided the number of years of schooling she had completed, ranging from 0 to 16 years. this number was divided by 2.	0-8
Income	Monthly household income was converted to US dollars using the exchange rate from January 1, 2010. Income was divided into octiles using the following scores and cutoffs: 1 (0–26), 2 (26.01-47), 3 (47.01-72) , 4 (72.01-106), 5 (106.01-135), 6 (135.01-200), 7 (200.01-293), 8 (293+).	0-8
Total	Scores in water and sanitation, assets, mother's education, and income were summed then divided by 32	0-1

Adapted demographic and Socioeconomic status survey (SES) the most widespread approach to direct measurement of household wealth is that used questions from the most recent by the Demographic and Health Surveys (DHS) questionnaires, Improved water and sanitation were based on World Health Organization definitions. Water and sanitation (W) component using 4 for improved and 0 for unimproved values.
